# Physicochemical Investigation of Psoralen Binding to Double Stranded DNA through Electroanalytical and Cheminformatic Approaches

**DOI:** 10.3390/ph13060108

**Published:** 2020-05-28

**Authors:** Douglas Vieira Thomaz, Matheus Gabriel de Oliveira, Edson Silvio Batista Rodrigues, Vinicius Barreto da Silva, Pierre Alexandre dos Santos

**Affiliations:** 1 Faculty of Pharmacy, Federal University of Goias, Goiania-GO 74605-170, Brazil; matheusgabriel06@hotmail.com (M.G.d.O.); edson.silvio.b@gmail.com (E.S.B.R.); 2 Department of Pharmacy, Pontifical Catholic University of Goias, Goiania-GO 74175-120, Brazil; viniciusbarreto.farmacia@gmail.com

**Keywords:** voltammetry, intercalating agent, furanocoumarin, thermodynamic, kinetics

## Abstract

This work showcased the first physicochemical investigation of psoralen (PSO) binding to double stranded DNA (dsDNA) through electroanalytical methods. Results evidenced that PSO presents one non-reversible anodic peak at electric potential (*E*_pa_) ≈ 1.42 V, which is associated with its oxidation and the formation of an epoxide derivative. Moreover, PSO analytical signal (i.e., faradaic current) decreases linearly with the addition of dsDNA, while the electric potential associated to PSO oxidation shifts towards more positive values, indicating thence that dsDNA addition hinders PSO oxidation. These findings were corroborated by the chemoinformatic study, which evidenced that PSO intercalated noncovalently at first between base-pairs of the DNA duplex, and then irreversibly formed adducts with both DNA strands, leading up to the formation of a cross-link which bridges the DNA helix, which explains the linear dependence between the faradaic current generated by PSO oxidation and the concentration of DNA in the test-solution, as well as the dependence between *E*_p_ and the addition of dsDNA solution. Therefore, the findings herein reported evidence of the applicability of electroanalytical approaches, such as voltammetry in the study of DNA intercalating agents.

## 1. Introduction

**Figure 1:**
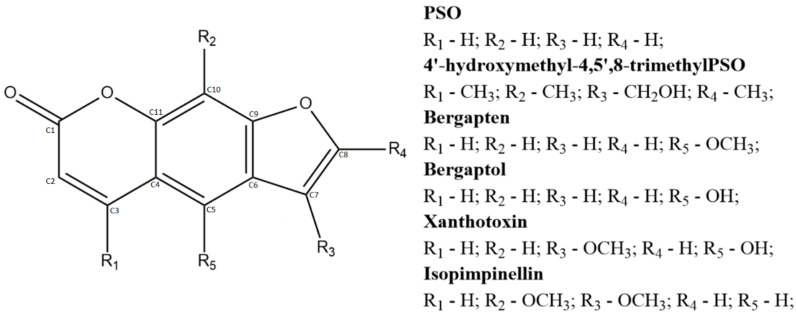
Linear furanocoumarins and their structures, namely: psoralen (PSO); 4’-hydroxymethyl-4,5’,8-trimethylpsoralen; bergapten; bergaptol; xanthotoxin; and isopimpinellin.

Psoralen (PSO) is a photosensitizing linear furanocoumarin derivative, whose biologic potential was therapeutically explored to treat cutaneous conditions such as psoriasis and vitiligo, as well as some forms of cancer [1,2,3,4,5]. This compound is a secondary metabolite of several plant families (e.g., Fabaceae, Moraceae, Rutaceae, etc.), and its chemical structure is the building block of all linear furanocoumarin derivatives, whose healthcare uses can be either allopathic or folk medicine-based [6,7,8]. Although the therapeutic use of furanocoumarins is well reported, the exact mechanisms of action regarding their many biological effects are still unknown. Some authors reported PSO interactions with immunomodulatory targets, while other reports shed light on the molecular mechanisms involved in its photochemical sensitizing effects [9,10]. Nonetheless, furanocoumarin chemical structures are relatable to several small ligands, which may confer such a wide range of possible biological targets (Figure 1).

Despite the acknowledged biological activities of furanocoumarin derivatives such as PSO, many reports attribute remarkable deoxyribonucleic acid (DNA)-binding properties to this compound, which may be involved in its therapeutic effects [11,12,13,14,15]. In this sense, several authors investigated PSO–DNA binding through spectrophotometric methods, as well as chemoinformatic approaches, resulting thence in the understanding that covalent linking is involved in PSO anchorage on DNA strands through PSO-Thymine adducts [11,13,15]. Considering that the contemporary models for PSO–DNA bonding involve the formation of a central cyclobutanyl moiety between the PSO-Thymine adduct, the overall reaction is a reductive-oxidative process whose thermodynamics and kinetics could foment a better understanding regarding drug–DNA interactions.

Although spectrophotometry is a reproducible and viable method for studying the physicochemical properties of drug–DNA interactions, such as those of PSO, alternative approaches such as electroanalytic investigation may provide valuable information regarding the redox processes therein involved [16,17,18]. In this context, methods such as voltammetry may shed light on several redox processes which drugs may undergo upon exposure to shifts in electric potential, as well as foment a better understanding of drug–DNA interactions involving electron transfer.

Therefore, considering PSO relevance as a model compound for drug–DNA interactions, and the importance of using complementary approaches to better understand its biological activities, this work is aimed at the characterization of PSO electrochemical behavior in presence of commercial calf-thymus double stranded (ds) DNA, and the exploration of the electroanalytical findings using available in silico models.

## 2. Results

### 2.1. PSO Redox Behavior without DNA

**Figure 2:**
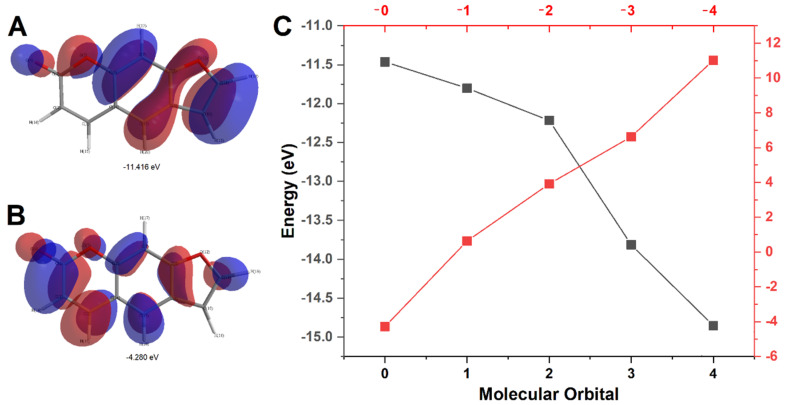
Graphical rendering of the first state (*n* = 0) of the highest occupied molecular orbital (HOMO) (**A**) and lowest unoccupied molecular orbital (LUMO) (**B**) for psoralen molecule. Positive charges are rendered in red while negative charges are rendered in blue. (**C**) Plot of molecular orbital (HOMO in lower *x*-axis and LUMO in upper *x*-axis) versus their respective energies in eV for psoralen and its oxidation product.

**Figure 3:**
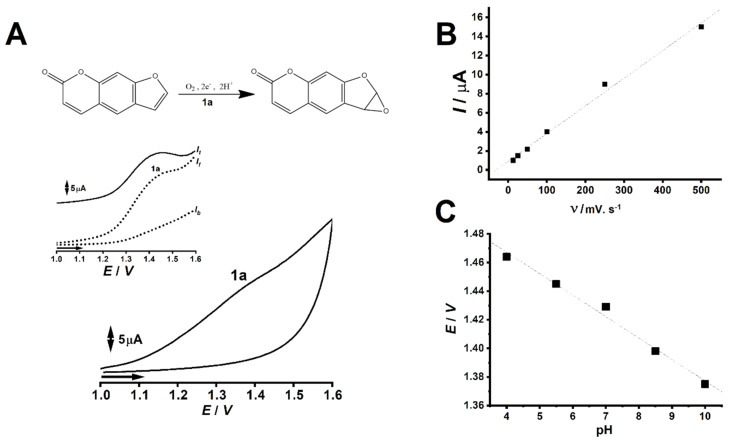
(**A**) Cyclic and Square Wave voltammograms showcasing the anodic peak (1a), which is associated to psoralen oxidation. The depicted CV was performed at 500 mV s^−1^. (**B**) Plot of anodic peak 1a faradaic current (*I*) vs. scan rate (ν) (r^2^ = 0.9932). (**C**) Plot of pH vs. electric potential associated to peak 1a (*E*) (r^2^ = 0.9874). All assays were performed in 0.1 mol L^−1^ PBS solution, pH 7.0, and Ag/AgCl/KCl_sat_ as reference electrode.

The first step of this investigation involved the rendering of highest occupied molecular orbital (HOMO) and lowest unoccupied molecular orbital (LUMO) models for PSO using Hückel approximation. Considering the abundance of π electrons as well as the sp^2^ hybridization of all carbons in PSO structure, this model offers trustful data for the proposition of possible sites for PSO oxidation as well as for the investigation of the transmission of the inductive effect over the entire molecule [19,20,21,22]. Data was collected until HOMO−4 and LUMO+4. Moreover, PSO redox behavior at a glassy carbon electrode was investigated through cyclic voltammetry (CV), square wave voltammetry (SWV), and differential pulse voltammetry (DPV). Results are depicted in Figure 2 and Figure 3.

### 2.2. PSO-dsDNA Redox Study

**Figure 4:**
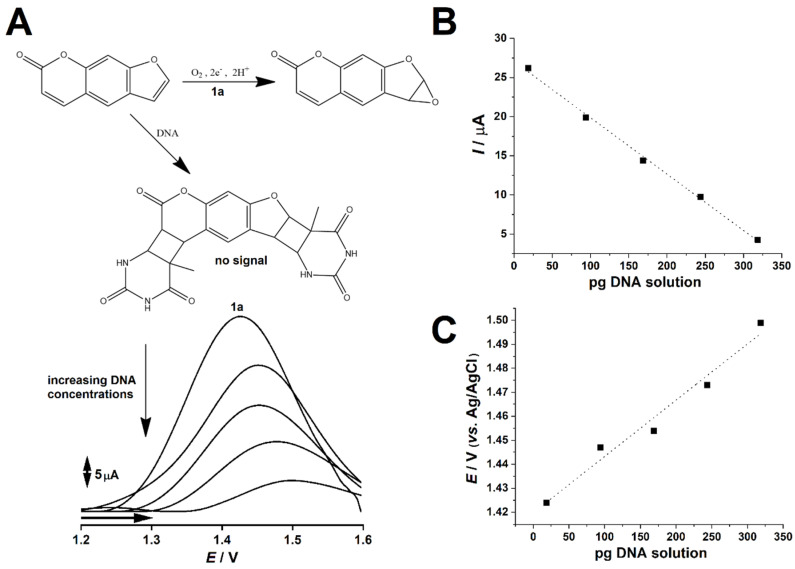
(**A**) Differential pulse voltammogram showcasing the effect of DNA addition on the anodic peak (1a) which is associated to psoralen oxidation. DNA-bound psoralen is non-electroactive, thence showcasing no anodic peak. (**B**) Plot of anodic peak 1a faradaic current (*I*) vs. the amount of added DNA (r^2^ = 0.9973). (**C**) Plot of electric potential associated to peak 1a (*E*) vs. the amount of added DNA (r^2^ = 0.9712). All assays were performed in 0.1 mol L^−1^ PBS solution, pH 7.0, and Ag/AgCl/KCl_sat_ as reference electrode.

In order to investigate the physicochemical changes associated with PSO binding to dsDNA, several DPVs were conducted at 1 mmol L^−1^ PSO solution pool under increasing proportion of 0.75 µg L^−1^ dsDNA solution, which ranged from 25 µL to 425 µL. The final volume of all test solutions were 1 mL. Results are depicted in Figure 4.

### 2.3. Description of PSO-dsDNA Interactions

**Figure 5:**
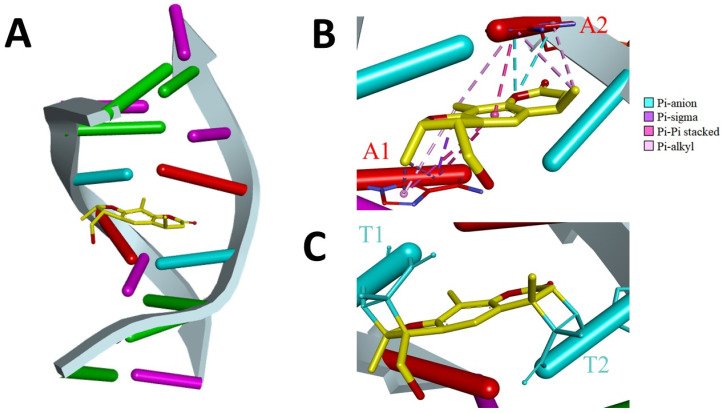
(**A**) Model of the cross-linkage between psoralen and DNA. (**B**) Adenine bases stacked coplanar with psoralen. (**C**) Psoralen covalently attached to thymines through a cyclobutane ring. The figure was generated with Discovery Studio v.2019.

In order to better explore PSO-dsDNA interaction, an investigation on the factual representations of the binding between these molecules was performed. Therefore, previously published nuclear magnetic resonance and restrained molecular dynamics results of PSO-dsDNA interaction were investigated [22]. The findings were segregated into two major approaches, namely: the electrostatic interactions between PSO and dsDNA residues in its vicinity, and the formation of covalent bonds between PSO-Thymine residues through a central cyclobutanyl moiety. Results are showcased in Figure 5.

## 3. Discussion

The rendered model evidenced that PSO’s first HOMO state showcased higher charge density around the furan ring (Figure 2A), thus suggesting that this moiety may exhibit higher thermodynamic proneness to undergoing oxidation [19]. Moreover, the LUMO surfaces concentrated around the pyrone moiety (Figure 2B), and the plot of molecular orbital versus their respective energies in eV showcased that for both compounds, LUMO charge shifted towards positive values, while HOMO shifted towards negative values (Figure 2C). This is an expected trend, given that higher states involve higher shifts in energy when redox processes are concerned, as showcased in a previous report [19].

The variation between HOMO and LUMO energies in eV also provides valuable information regarding the transmission of inductive effects through the molecule, as Hückel approximation is quite reproducible when planar and π electron-rich compounds are concerned [20]. Although differential functional theory is a more common method for investigating redox thermodynamics at quantum level, the particularities in PSO structure makes simpler approaches feasible. Nonetheless, Hückel calculations concerning PSO’s HOMO-0 and LUMO-0 showcased a higher energy gap than further stages up to *n* = 3, which suggests that up until the first electron transfer (i.e., oxidation or reduction), the inductive effect of electronegative moieties will be transferred through the molecule following the theory of aromaticity, as well as the principle of vinylogy [20,23,24].

Electrochemical results showcased that under CV and SWV, PSO presents one non-reversible anodic peak at *E*_pa_ ≈ 1.42 V, which correlates to the oxidation of electroactive moieties in its molecule (Figure 3A) [25,26]. Moreover, given the linearity of the plot of anodic peak 1a-faradaic current (*I*_p_) vs. scan rate (ν), an adsorption-controlled electrochemical reaction can be suggested for the first oxidative process (Figure 3B) [19]. Furthermore, the linearity showcased by the plot of pH vs. electric potential associated to peak 1a (*E*_p_) suggests an equivalence of protons and electrons for anodic process 1a (Figure 3C) [19,27].

Given PSO’s status as major furanocoumarin marker alongside bergapten, this phytochemical is widely studied. Many authors investigated PSO’s stability against oxidative degradation through different approaches, and its voltammetric characterization was also described in literature [28,29,30]. Although distinct oxidation pathways were suggested for this chemical, most reports rely on the formation of a furan epoxide derivative upon irreversible electric potential-mediated oxidation, which involves equivalence of electrons and protons [28,29]. Nonetheless, this oxidative pathway was also described for PSO cytochrome-mediated catalysis, which also renders a furan epoxide metabolite [30].

Considering that HOMO-0 modeling showcased the localization of most negative charges on the furan moiety of PSO (Figure 2A), it can be strongly suggested that this moiety does undergo oxidation at the first anodic process [19]. Notwithstanding, the equivalent involvement of protons and electrons for the synthesis of the furan epoxide derivative further supports an oxidation pathway contemplating this reaction product. Nonetheless, the linearity between electric potential and pH strongly hints the equivalent number of protons and electrons involved in PSO first oxidation (Figure 3C) [27].

Literature reports that conjugated aromatic systems are prone to undergo electro-adsorption on carbon-based electrodes [31,32,33,34], as this effect is usually observed in plant secondary metabolites of the phenylpropanoid pathway. In this sense, given PSO’s nature as a furanocoumarin secondary metabolite of this very same metabolic route, several CVs were assayed under increasing scan rates in order to assess if its oxidation was adsorption or diffusion controlled.

Results showcased linearity between electric current and scan rate (Figure 3B), which thence suggests an adsorption-controlled electrochemical reaction [19,35]. Nonetheless, these findings were in consonance to literature regarding PSO electro-oxidation at carbon-surfaces, and its implications were also perceptible mid-analysis, hence strong electrode fouling after the first scans as well as peak broadening and deformation. Due to this phenomenon, we adopted an extended electrode-surface cleaning protocol involving physical abrasion with alumina solution, followed by electrode conditioning at buffer solution under 10 CV scans between 0 to 1.2 V at 50 mV s^−1^.

Results showcased that increasing concentrations of dsDNA led PSO faradaic signal to decrease. This finding suggests that PSO binding to dsDNA does not involve electron transfer on the electrode surface. The possible involved reaction is showcased in Figure 3A. Moreover, PSO faradaic signal steadily decreased upon increasing dsDNA concentrations, showcasing a linear trend in the interval herein studied (Figure 4B). In addition, PSO’s oxidation thermodynamics seemingly changed upon the presence and increasing concentrations of dsDNA, as suggested by the regular shifts of electric potential towards positive values (Figure 4A), as well as their linearity, according to the increase of dsDNA concentration (Figure 4C).

PSO–dsDNA binding is well reported in literature, and its kinetics and thermodynamics were thoroughly studied under spectrophotometric approaches, as the decrease of PSO absorbance values were a common finding [11]. Nonetheless, several reports showcased the decrease of the absorbance of DNA-coupling agents upon addition of DNA, which suggested that the physicochemical properties of these chemicals might change upon binding to DNA, thus reducing the observed signal [36,37,38,39]. Our results were in consonance to literature, given that the PSO electrochemical signal decreased linearly with the increase of dsDNA concentrations (Figure 4A,B).

PSO electrochemical oxidation at glassy carbon electrode surfaces occurs at *E*_pa_ ≈ 1.42 V when the voltammetric assay is carried out in 0.1 mol L^−1^ PBS solution, pH 7.0. However, the presence of dsDNA in the test solution may lead to the anchorage of PSO to dsDNA through PSO-Thymine adducts [15]. Given that this reaction seemingly does not lead to electron transfer on the electrode surface, only non-bound PSO was available to undergo oxidation, which explained the signal decrease as well as linearity associated to increasing dsDNA concentrations (Figure 4B) [18]. Notwithstanding, the linear increase of PSO’s oxidation thermodynamic parameter (i.e., *E*_pa_) hints that increasing dsDNA concentrations hinders non-bound PSO oxidation at the electrode surface, thence suggesting PSO proneness to binding to dsDNA, which is nonetheless widely reported to DNA-intercalating agents [18].

The model for the cross-linkage between PSO and dsDNA showed that the twofold symmetry of the DNA duplex was broken by PSO adduct formation (Figure 5A). PSO initially intercalates noncovalently between base-pairs of the DNA duplex which may stabilize its structure due to charge delocalization, as well as lead to steric hindrance [12,13,15], thus hindering electro-oxidation and increasing the electric potential associated with peak 1a, as seen in Figure 3. The adenine bases are stacked coplanar with PSO, and this arrangement allows electrostatic (Pi-anion) and hydrophobic (Pi-sigma, Pi-Pi-stacked, and Pi-alkyl) interactions (Figure 5B) [40]. On the other hand, thymine is the primary nucleoside in dsDNA at which reaction occurs [15]. The thymine bases were in cis-syn geometry relative to PSO, and the reaction takes place between the carbon atoms C5 and C6 of the thymines bonded to carbon atoms C4 and C3 of the pyrone ring, and to carbons C4′ and C5′ of the furan ring of PSO, thus leading to the formation of the cyclobutanyl adduct [15,40,41]. The covalent structure of the cross-link fixes the orientation and position of thymines and bridges the helix (Figure 5C), moreover this bonding is non-reversible without photolyase enzymes, which therefore explains the linear dependence of the faradaic current generated by PSO oxidation and the concentration of DNA in the test solution.

## 4. Materials and Methods

### 4.1. Solutions and Reagents

PSO (Sigma) was diluted in purified water (conductivity ≤0.1 µS.cm^−1^) obtained from Milli-Q purification system Millipore S/A (Molsheim, France), in order to render stock solutions of 0.01 mol L^−1^. Potassium chloride, disodium hydrogen phosphate, potassium hydrogen phosphate, and sodium chloride were used to prepare a 0.1 mol L^−1^ phosphate buffered saline (PBS) solution, pH 7.0. Furthermore, 1.5 g of calf-thymus dsDNA was diluted in 2 mL PBS, pH 7.0, in order to render a concentrated mixture for the studies.

### 4.2. Electrochemical Assays

Voltammetric experiments were carried out in a potentiostat/galvanostat Autolab III^®^ integrated with the GPES 4.9^®^ software, Eco-Chemie, Utrecht, Netherlands. The measurements were performed in a 1.0 mL one-compartment/three-electrode system electrochemical cell consisting of a glassy carbon electrode of 6.28 mm^2^ area, Pt wire, and Ag/AgCl/KCl_sat_ electrode (Lab solutions, São Paulo, Brazil), representing the work electrode, the counter electrode, and the reference electrode, respectively. The work electrode was subjected to thorough cleaning with alumina (Arotec) solution and rinsing with water after each experiment to ensure surface cleaning from oxidation products. An electrode cleanse was ensured by blanc CV-assays conducted in an electrochemical cell containing support electrolyte solution (phosphate saline solution, pH 7.0).

Experimental conditions for Cyclic Voltammetry (CV) were scan rate (υ) of either: 12.5, 25, 50, 100, 250, or 500 mV s^−1^ (adsorption study), and of 100 mV s^−1^ for preliminary redox profiling. The experimental conditions for Square Wave Voltammetry (SWV) were pulse amplitude of 50 mV, frequency of 50 Hz, and potential increment of 2 mV. The experimental conditions for Differential Pulse Voltammetry (DPV) were pulse amplitude of 50 mV, pulse width of 0.5 s, and scan rate of 10 mV s^−1^.

All CV, SWV and DPV experiments for PSO redox investigation employed a scan range between 1.0 to 1.6 V, while PSO–dsDNA interaction was studied using DPV, whose scan range was from 1.6 to 1.8 V. Moreover, all voltammetric assays were performed in triplicates, and results were expressed as a mean of triplicates. The electrochemical cell employed 0.1 mol L^−1^ PBS solution, pH 7.0, as support electrolyte and data were analyzed and treated with Origin 9b^®^ software.

### 4.3. PSO Redox Behavior without DNA

In order to investigate PSO redox behavior without dsDNA addition, CV was conducted to preliminarily investigate the electric potentials (thermodynamic parameter) which could induce PSO oxidation. Thereafter, several CV scans were conducted at varied scan rates in order to investigate if PSO electrochemical oxidation was adsorption or diffusion controlled.

Considering the linear sweep provided by CV, the evaluation of the slope between the faradaic signal and the scan rate was more easily achievable than through other voltammetric techniques, readily providing information regarding the dependence of electro-oxidation to diffusion or adsorption processes. Although effective for this purpose, the linear sweep of the electric potential offered by CV takes a considerable amount of time to be performed, which enhances the adsorption of oxidized species on the electrode surface and might lead to artifacts, such as peak broadening and deformation.

Regarding the use of SWV, this technique was conducted to evaluate possible reversibility of PSO oxidized states. This technique was selected for its fast performance, which provides less time for the adsorption of oxidized species on electrode surfaces, as well as for the fact that the square wave signal allows the simultaneous visualization of both anodic and cathodic processes, which provides more reliable information on redox reversibility than linear sweeps, such as those of CV.

Furthermore, DPV was conducted using several buffers of varied pH values in order to estimate if the redox processes involved an equivalence of protons and electrons. DPV electric potential sweep is somewhat similar to that of SWV, however the square wave signal is shaped into a differential pulse pattern. This allows enhanced signal gathering when faradaic processes are concerned. On the other hand, this technique is lengthy, which results in higher influence of capacitive currents, as well as oxidized species adsorption on electrode surfaces, thence leading to the deformation of the voltammogram.

### 4.4. PSO-dsDNA Redox Study

The study of PSO redox features in the presence of dsDNA was performed through DPV assays of several PSO solutions upon addition of varied volumes of 0.75 µg L^−1^ dsDNA solution. The final PSO concentration was kept constant for all assays, namely 1 mmol L^−1^ PSO in a total of 1 mL of test solution. The added volumes of 0.75 µg L^−1^ dsDNA solution ranged from 25 µL to 425 µL, with an increment of 100 µL for each sampling. All experiments were conducted at 25 °C under direct illumination from commercial 110 W fluorescent lights (Phillips) and diffused sunlight.

### 4.5. Description of PSO Oxidation and PSO-dsDNA Interactions

Given the dependence of the oxidative processes to electron shifts between orbitals [19], we employed Hückel calculations to render the highest occupied molecular orbital (HOMO) and lowest unoccupied molecular orbital (LUMO) models of PSO and its proposed oxidation product, in order to gather information about charge distribution along its molecule [42]. These calculations were performed after energy minimization procedures, which involved force field approaches from classic molecular mechanics (MM2) and assisted model building and energy refinement (AMBER) tools. All calculations were performed on Chem3D^®^ Software and UCSF Chimera software (version 1.13.1) employing standard commands.

In order to better correlate the electroanalytical findings to the binding models described in literature, the software Discovery Studio^®^ (BIOVIA) version 2019 was used to visualize and render electrostatic interaction models of previously published nuclear magnetic resonance results of 4’-hydroxymethyl-4,5’,8-trimethy-PSO interaction with dsDNA, which were retrieved from Protein Data Bank (PDB) (PDB code 204D) [22].

## 5. Conclusions

This work showcased the first physicochemical investigation of PSO linking to dsDNA through electroanalytical methods. Results evidenced that PSO presents one non-reversible anodic peak which is associated with its oxidation and the formation of an epoxide derivative. Moreover, the PSO analytical signal (i.e., faradaic current) decreases linearly with the addition of dsDNA, while the electric potential associated with PSO oxidation shifts towards more positive values, indicating thence that dsDNA addition hinders PSO oxidation. These findings were corroborated by the chemoinformatic study, which evidenced that PSO binding leads to the formation of a cross-link which bridges DNA helix, explaining the linear dependence between the faradaic current generated by PSO oxidation and the concentration of DNA in the test solution, as well as the dependence between *E*_p_ and the addition of dsDNA solution.
